# Application of unmanned aerial vehicle (UAV) thermal infrared remote sensing to identify coal fires in the Huojitu coal mine in Shenmu city, China

**DOI:** 10.1038/s41598-020-70964-5

**Published:** 2020-08-17

**Authors:** Xiaoyuan He, Xingke Yang, Zheng Luo, Tao Guan

**Affiliations:** 1 School of Earth Science and Resources, Chang’an University, Xi’an, 710054 China; 2 Aerial Photogrammetry and Remote Sensing Bureau of China National Administration of Coal Geology, Xi’an, 710199 China

**Keywords:** Environmental sciences, Fossil fuels

## Abstract

China is a major coal-producing country that consumes large amounts of coal every year. Due to the existence of many small coal kilns using backward mining methods, numerous worked-out areas have been formed. The coal mines were abandoned with no mitigation, so air penetrates into the roadways and contacts the coal seams; as a result, the residual coal seams spontaneously ignite to form coal fires. These coal fires have burned millions of tons of valuable coal resources and caused serious environmental problems. To implement fire suppression more effectively, coal fire detection is a key technology. In this paper, thermal infrared remote sensing from unmanned aerial vehicle combined with a surface survey is used to identify the range of coal fires in the Huojitu coal mine in Shenmu city. The scopes and locations of the fire zones are preliminarily delineated, which provides an accurate basis for the development of fire suppression projects.

A coal fire is a subsurface phenomenon that causes not only losses of valuable natural resources but also environmental problems, such as surface cracks, subsidence and collapse, and atmospheric pollution, eventually endangering human security^1–3^. The term coal fire refers to a spontaneous combustion phenomenon in which a coal body is in contact with air and oxidizes to burn under natural conditions that occur in exposed coal seams or underground, so as in coal waste and storage piles^4^. Coal fires occur everywhere such as the United States, South Africa, India, Australia, Indonesia, China and Canada^4,5^, which are major coal-producing countries. With plenty of thick and shallow coal beds, underground coal fires are burning in Ningxia, Xinjiang, Inner Mongolia and Shaanxi provinces in Northern China^6^. The fires in China are usually triggered by human interference^7^ because human alteration of the natural environment of coal seams makes them easier to oxidize and spontaneously combust. The Jurassic coalfield is located in the six counties, namely, Dingbian, Fugu, Hengshan, Yuyang, Jingbian and Shenmu in Yulin city in northern Shaanxi; it covers 27,000 km^2^ in area and contains 138.8 billion tons of proven reserves of coal resources^8^. Jurassic coalfields in northern Shaanxi still have a large amount of coal resources, and burnt rocks(clinkers) are widely distributed during the geo-historical period^9^; these rocks have been baked or melted by the burning of underlying coal beds, providing evidence of past coal bed fires^10^ and showing that conditions are suitable for spontaneous combustion in this area. In recent years, with a large number of developed coal resources, human activities change the environment and thus improving the chance of spontaneous coal combustion, the Jurassic coal fields in northern Shaanxi have caused many surface and underground coal fires, mainly distributed in Shenmu, such as in the Longyan, Tanyaoqu, and Huojitu coal mines (HCM) (Fig. 1b).

**Figure 1:**
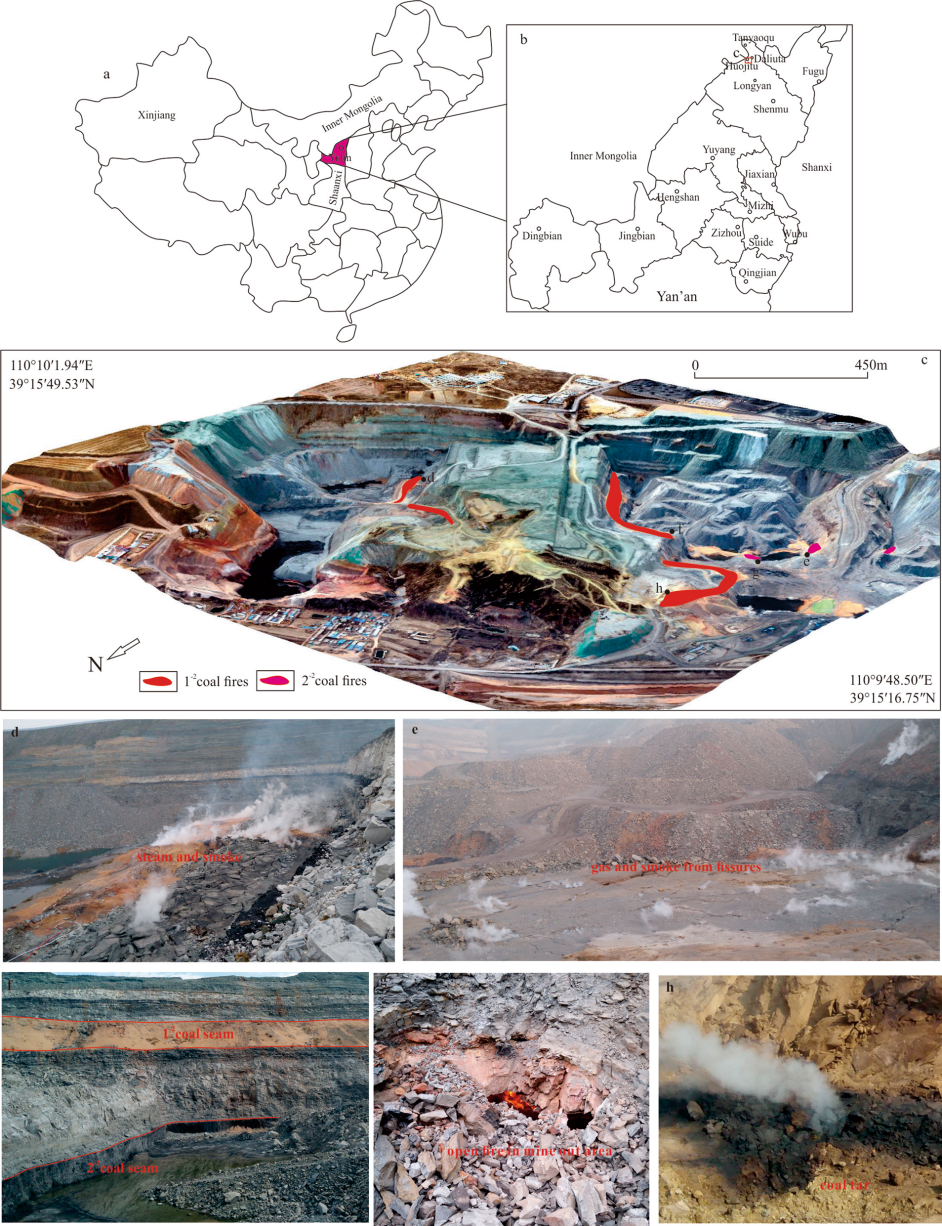
The location of the HCM: (**a**) shows the location of Yulin city in China. (**b**) show the HCM located in the Shenmu city; (**c**) is a three-dimensional based on digital orthophoto map (DOM) and digital surface model (DSM) that are acquired on 16 February 2019, red and magenta bands indicate 1^−2^ and 2^–2^ coal fire respectively. (**d**)–(**h**) ground survey, (**d**) and (**e**) are gas and smoke are emitted along the surface fissures.(**f**) is 1^−2^ and 2^–2^ coal seams in outcrop, 1^–2^ coal seam covered by loess for extinguishing fire. (**g**) is an open fire in mine-out area. (**h**) is coal tar from vent.

Accurate detection of the coal fire combustion centre, range and depth is a major problem for coal fire exploration technology, and it is also the basis of coal fire suppression projects. It is difficult to determine the extent of underground coal fires because of a few surface factors, including vegetation, rock and soil cover over the coal bed^11^. At present, principal detection methods include remote sensing, borehole temperature measurements, and geochemical and geophysical measurements^12^. Since the early 1960s, remote sensing has become a convenient and useful tool for the monitoring and detection of coal fires based on surface temperature anomalies^11,13–17^^.^ Interferometric synthetic aperture radar (ISAR) can detect subtle surface deformation but the subsidence may not always equal to an active fire region^18^. Several researchers have used magnetic methods to characterize underground coal fires^3,16,19–27^. Magnetic surveys provide high-resolution coal fire mapping and delineate previously burned, active coal fire and non-coal fire regions, and the magnetic properties of materials change with temperature^16^. To date, only a few geophysical or remote sensing methods have been applied for coal fire detecting in Shaanxi Province, but there are some problems. For example, due to the destruction of the original terrain, a magnetic exploration line cannot be measured through an active coal fire or artificial cliff, or the scattered distribution of highly magnetic burnt rock from other places on the surface affects the results obtained by the magnetic method, so the magnetic method cannot effectively delineate the coal fire boundary. Radon measurements are not possible or are not accurate due to surface fissures and detached loess. In the past, airborne thermal infrared (TIR) remote sensing had been suitable for the identification of large fires, but small-scale mines are not easy to identify, or the data are not precise enough. Although providing high temporal and spatial resolution, imaging systems mounted on manned airborne platforms are limited by high operational complexity and costs^28^. The development of unmanned aerial vehicle (UAV) remote sensing with low-altitude level has the characteristics of high spatial resolution, frequency and cost performance, and can complement satellite remote sensing capabilities, alleviating the contradiction between high spatial resolution and temporal resolution^29,30^. This method provides the safe and rapid probe of thermal areas, often occur in dangerous or inaccessible terrain^31–32^. In this paper, due to the destruction of the original landforms and interference factors, geophysical surveys are not available in fire zones, so low-altitude UAV-based TIR remote sensing, ground surveys, and boreholes are used to comprehensively detect and delineate coal fire areas in the Huojitu coal mine (HCM) in Shenmu city, northern Shaanxi.

The HCM is located approximately 4.5 km west of Daliuta town, Shenmu city, Shaanxi Province (Fig. 1a), between N 39° 15′ 16.75″–N 39° 15′ 49.53″ and E 110° 9′ 48.50″–E 110° 10′ 1.94″; it has a typical arid continental monsoon climate covering an area of 2.71 km^2^. From east to west, the length of the mining area is approximately 600–2,500 m, and north to south, the width is approximately 300–1,500 m, with most of the original landforms altered. The area is located on the northern Shaanxi slope of the Ordos Basin, a monoclinic structure inclined to the northwest. The strata are gentle and nearly horizontal at 1°–3°, and there is no fault or magmatic activity.

The coal seams in HCM are mined in the Middle-Lower Jurassic Yan'an Formation (J_1–2_*y*), the only exposed unit in the study area. In the regional area, the Yan'an Formation (J_2_*y*) is separated by disconformities from the underlying Upper Triassic Yongping Formation (T_3_*y*) and the overlying Middle Jurassic Zhiluo Formation (J_2_*z*). The thickness of the Yan'an Formation is generally between 260 and 316 m and is divided into five sections, each of which contains a coal group, and the coal groups are numbered 1–5 in order from bottom to top (Fig. 2). The study area is covered mainly by sandstones, siltstones, shales and coals. The major mining in the HCM occurred in the 1^–2^ and 2^–2^ coal seams; 1^–2^ has a thickness from 2.80 to 11.38 m with an average of 10.20 m, and the average elevation of the 1^–2^ coal floor is 1,073 m above sea level; 2^–2^ has a thickness from 4.48 to 5.25 m with an average of 4.75 m, and the floor elevation 1,037 m above sea level (Fig. 1f), the pillar and room method was adopted between 1985 and 2001. Opencast mining activity (2002–2011) often leads to bulk volume of mining wastes and large fresh rock surfaces^28^. From 1985 to 2011, due to years of random mining and excavation of small coal kilns and the typical dry natural climatic conditions, under which coal seams are prone to spontaneous combustion, the residual coal seams are oxidized and can be naturally ignited. Mining of the 1^–2^ coal on the east and west sides was completed, and a pit was formed with the lowest elevation at 1,040 m and residual 2^–2^ coal pillars in the middle stripper platform to be mined with the highest elevation at 1,156 m and residual 1^–2^ coal pillars; the mining formed steep terrain (an escarpment) with relief of approximately 116 m (Fig. 1c). According to a ground survey, the coal fire is mainly located on the escarpment wall where the coal seam was deeply excavated with a large-scale worked-out area, and copious gas and smoke are emitted along the surface fissures. The hot fumes reach temperatures as high as 340.0 °C, locally separating out coal tar. Sulfur and mirabilite are also found around some gas vents (Fig. 1d, e, h). This process occurs because the pit accelerates the flow of oxygen penetrating the combustion zone, thereby exacerbating the fire's spread to the west and east of the pit. The lower layer of coal belongs to the China Shenhua Group. With the low-rank coal beds rich in volatile matter and dry climate, as the rooms between pillars collapse and allow air to enter from the surface^10^, the coal in the HCM is prone to spontaneous combustion. The coal beds in the HCM are nearly horizontal, and when a fire starts, it first spreads along the outcrop. The Yulin Bureau of Natural Resources and Planning has carried out some fire-fighting measures in the HCM, such as loess cover and water injection, but the fires are still active, indicating that these measures have not been wholly successful.

**Figure 2:**
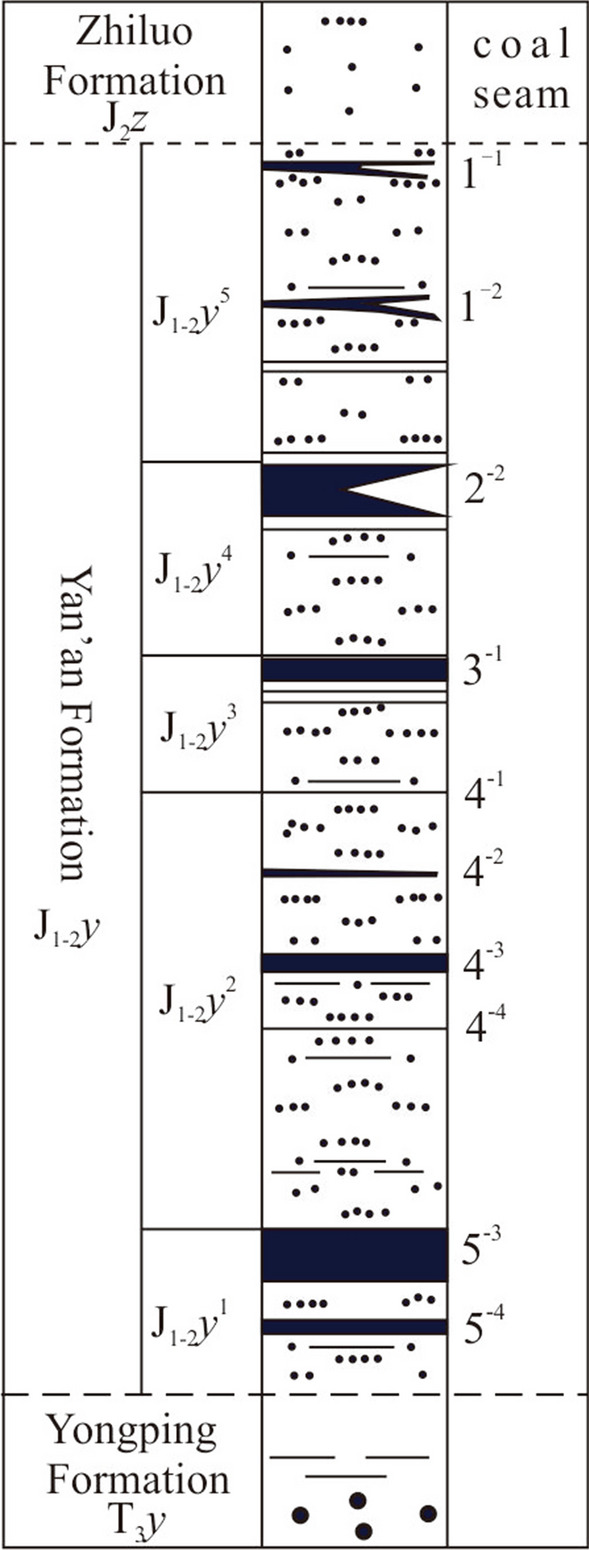
Schematic diagram of the numbering of coal seams in the Yan'an Formation.

## Methodology

TIR is a remote sensing method that detects variations in heat on Earth’s surface^34^. The use of airborne TIR for mapping and studying coal fires has greater resolution and availability than satellite TIR^11^ imagery. Recent advances in UAVs equipped with global positioning systems (GPSs) and digital cameras are reducing the cost of collecting imagery^30^. High-resolution thermal cameras have been successfully mounted on aircraft platforms and on UAVs, increasingly using high-performance sensors with smaller size and weight and greater spectral and spatial resolutions. The thermal cameras can reach centimetre-scale ground resolution and provide sufficient accuracy.

In our research, TIR Zenmuse XT2 cameras mounted onto a UAV DJI M210 were used to acquire data. The Zenmuse XT2 gimbal and cameras, which included a forward looking infrared detector and a visual camera, provided both infrared and visual images simultaneously. The forward looking infrared camera performed high-sensitivity infrared scanning at 640/30 fps and was equipped with an uncooled vanadium oxide (VOx) microbolometer to measure longwave radiation in the spectral range 7.5 ~ 13.5 μm and a temperature range of -20 to 135 °C (high gain); it had a 25 mm lens and acquired image frames of 640 × 512 pixels as raw 8-bit digital numbers (DNs) at the rate of less than 9 Hz. The visual camera captured 4 K videos and 12 megapixel photos (https://support.pix4d.com/hc/en-us). Several studies showed that TIR surveys conducted during the fall or predawn were best for detecting coal fires^35–38^, but that RGB orthophotos were best obtained during the day. To acquire both types of data simultaneously, the flight was carried out from 7 a.m. to 10 a.m. on 22 October 2019, which was a cloudy day; the heating caused by sunlight was very small, and solar radiation was negligible. As described previously literature, an appropriate flight plan was determined using the DJI Ground Station software^30^. The flight plan was then uploaded to the quadcopter’s flight controller using the DJI Vision App^30^. Accordingly, both in-flight navigation and image capture were autonomous^30^. The internal time of the camera was set to the GPS time prior to the flight to ensure that the images could be easily synchronized with the position data in the UAV GPS log file^28^. Ground control points (GCPs) measured with differential RTK GPS were established before the flight so that the resulting orthophoto imagery and digital elevation models (DEMs) could be accurately georeferenced and tested^30,32^. On a clear day with good visibility, the flight was conducted at a relatively low angle with respect to the horizon. Three flights were conducted with a flight altitude of 300 m, the frontal overlap rate was 75%, and the side overlap was 65% to obtain high-accuracy results. When there is high overlap between 2 images, the common area captured is larger, and the key points can be matched. Therefore, the main rule is to maintain high overlap between the images. The route length was approximately 21.6 km, and 431 overlapping images were processed using the Pix4D software (https://support.pix4d.com/hc/en-us). A digital orthophoto image and a digital surface model (DSM) of the mining area were generated, with a ground resolution of 3.8 cm.

**Figure 3:**
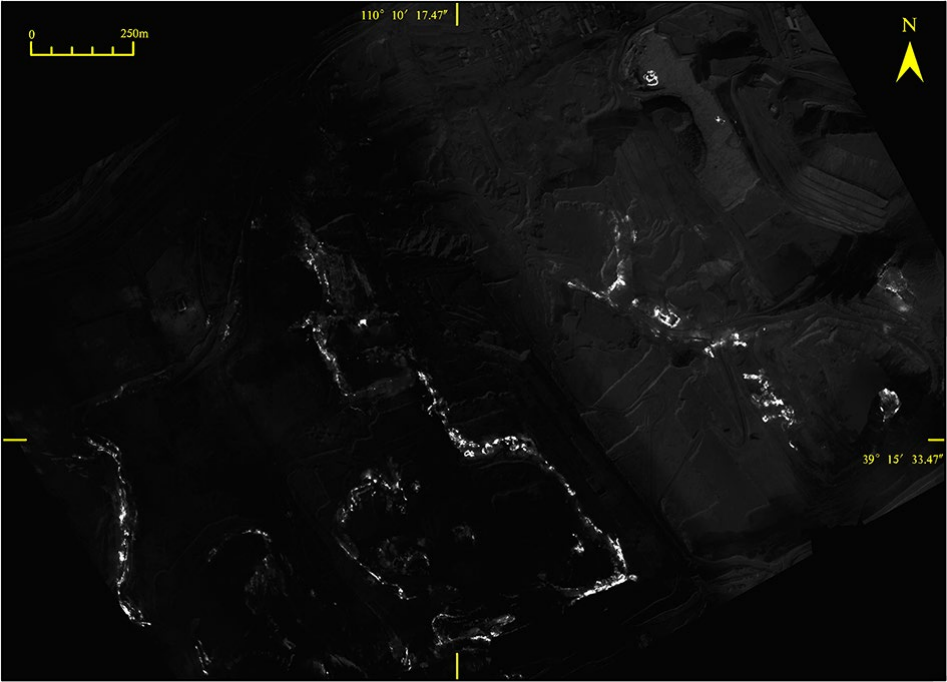
TIR image of HCM (Bright colors represent high temperature abnormalities).

A program was written in Python code to calculate the maximum and minimum grey values of the TIR image, which were used to invert the surface temperature, and the thermal anomaly distribution area caused by coal fires and the locations of fire area were accurately determined (Fig. 3), with a ground resolution of 40 cm (pixel size). During the flight, the ground temperature was measured simultaneously to obtain the key parameters for temperature calibration and inversion of the TIR image; the measurement used a TIR thermometer Fluke 62 Mini, with a temperature measurement range of -30 ~ 500 °C, and a temperature error of ± 2 °C (https://www.fluke.com). The original 8-bit raw DNs were collected by the TIR sensor, which in the thermal imagery represented at-sensor radiance. Matching pixels were extracted from the thermal imagery, and based on the matching reference temperatures, linear regression was calculated^28^. After mosaicking and co-registration, the DN values were converted to absolute temperature in °C based on an empirical line correction^28^, according to the radiation conduction equation and the Plank function. With this empirical relationship, we converted the whole thermal mosaic into absolute temperature, assuming a constant emissivity of 0.95^28^. A similar method can be found in the literature ^28,30,39^.

## Results

### UAV thermal infrared

Airborne TIR technology has a wide detection range and high image resolution, which provides great spatial detail for mapping coal fires^40–42^, especially based on UAVs. Thermal anomalies induced by underground coal fires can be extracted from TIR data using an exclusion method^43^, together with field temperature measurements. TIR data are widely used to delineate subtle surface thermal anomalies associated with underground coal fires^44^.

**Figure 4:**
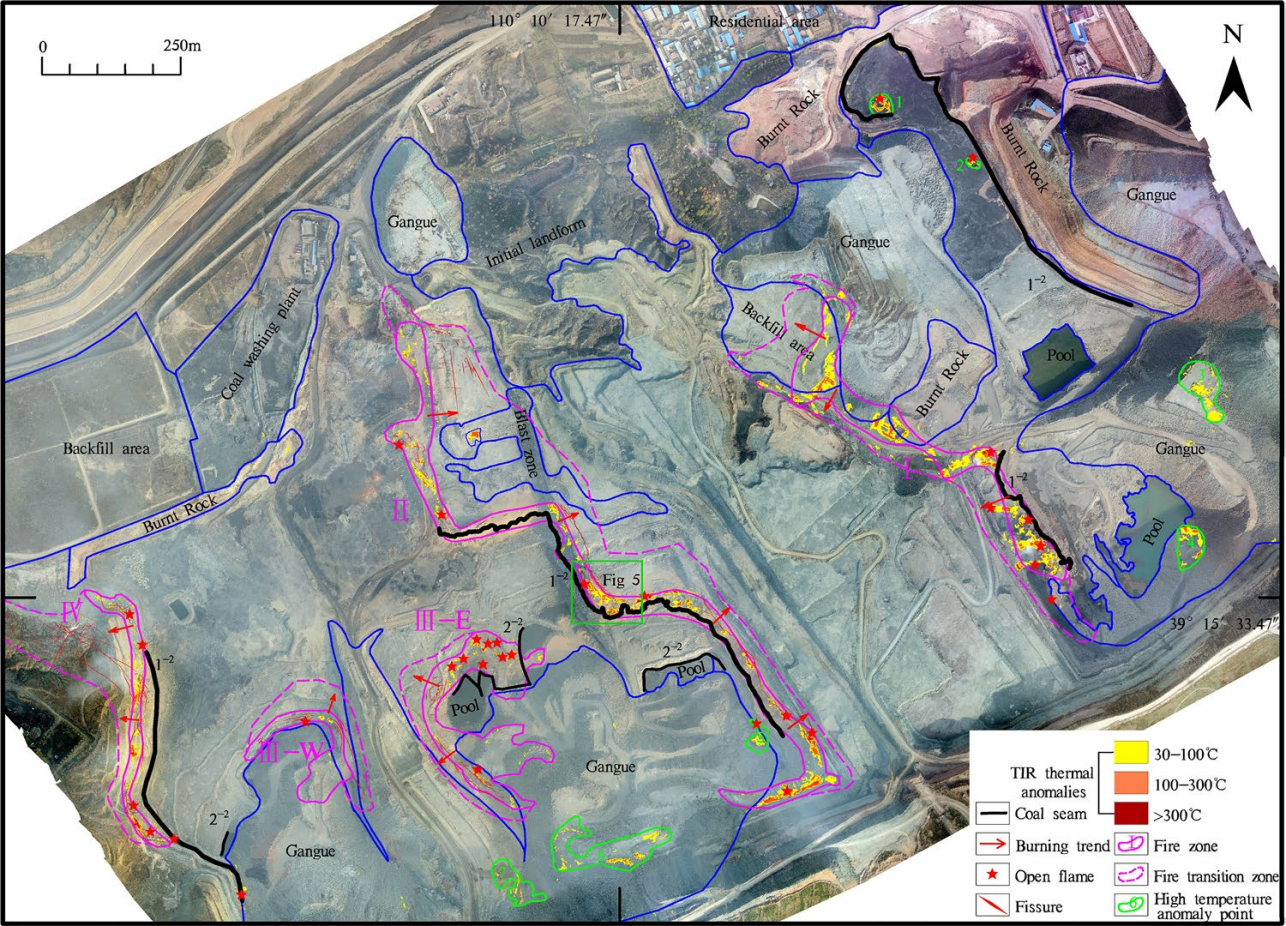
Digital orthophoto map (DOM) of the HCM and superimposed thermal anomaly. Divided into I, II, III, and IV four coal fire zones by thermal anomalies and surface survey. Green box show the location of Fig. 5.

**Figure 5:**
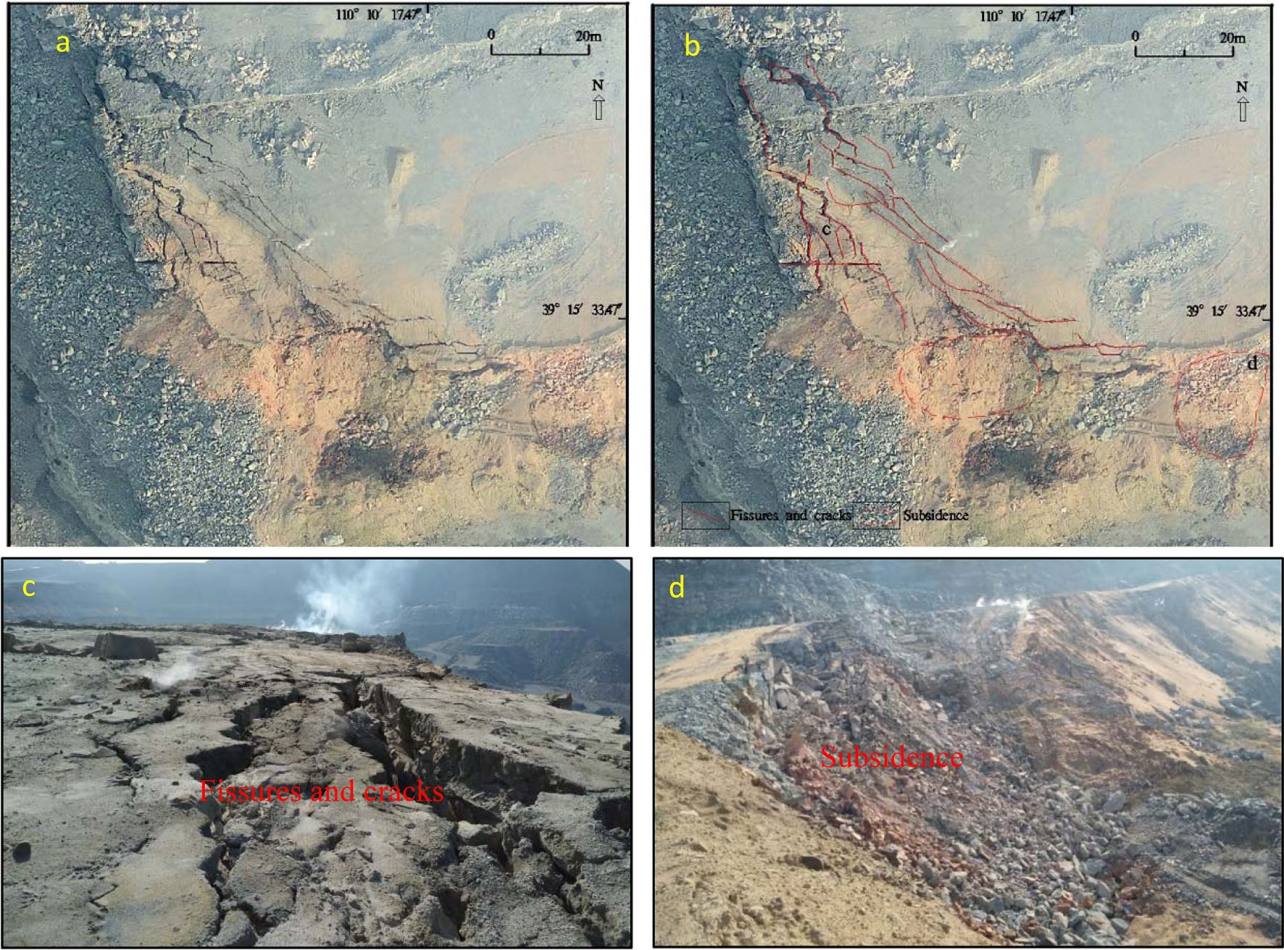
Fissures and subsidence in the fire zone (the location show in Fig. 4). **a**. DOM; **b**. Interpret fissures and subsidence on DOM; **c** and **d** field photos (Camera direction 158° and 95° respectively).

In our research, the digital orthophoto map (DOM) and the TIR image (Fig. 3) obtained after processing the raw data collected by the drone. The image was clear, and the colours were bright. The TIR data and the RGB orthophoto relied upon ground control points (GCPs) or orientation measurements from inertial measurement units (IMUs) to enable accurate georeferencing of the imagery. Then the thermal infrared DOM was registered with the RGB DOM, and the registration error was 0.4 pixels. From the DOM, the coal seams, fissures, burnt rocks, gangue, pool, backfill area, coal washing plant, initial landforms, residential areas, etc., could be clearly interpreted based on multi-scale segmentation and were verified with surface surveys (Fig. 4). Thermal anomalies in the fire zone are very obvious and appears as high-brightness spots or bands on the TIR remote sensing image. The difference in brightness reflects the temperature difference of the fire zone. The thermal anomaly packet is extracted and superimposed on the DOM to interpret four banded fire zones and seven small temperature anomalies (Tables 1, 2).The development of vents, cracks, subsidence on the surface result from underground coal fires. Such features sporadically extent in spatial and vary in dimension from a few to tens of metres^45^. Combining the investigation of surface fissures and cracks with or without smoke, and temperature measurements, the fire transition zone is delineated. Other areas are normal areas, which are non-fire zones. From east to west, the study area is divided into four coal fire zones I, II, III and IV; other fires are small and sparse, mostly burning gangue extending 30–100 m. The fire zone has the characteristics of high temperature, heat waves, flames, and new burnt rocks. It is the centre of the coal fire zone with many open fires according to the field survey (Fig. 4). Slumping land surface, large and wide fissures are evidently visible along the edges of the fire. Combustion leads to subsidence and many cracks (Fig. 5), which are very dangerous and basically make access difficult. In the fire transition zone, chimneys form in the fire zone, flames are basically not seen, fissures and cracks develop, and smoke is emitted from the cracks with high-temperature gas. This transition zone is the margin of the coal fire zone. The coal fire area refers to the cumulative area of fire zones and fire transition zones.

**Table 1:** Coal fires statistics.

Fire Number	Ignite coal seam	Thickness of coal(m)	Coal fire area (m^2^)	Fire zone area (m^2^)	Fire transition zone area (m^2^)
I	1^–2^	10.20	75,634.96	33,521.64	42,113.32
II	1^–2^	10.20	143,848.09	49,760.82	94,087.27
III-E	2^–2^	4.75	43,458.82	31,132.91	12,325.91
III-W	2^–2^	4.75	24,480.31	14,143.17	10,337.14
IV	1^–2^	2.80	46,037.48	11,573.49	34,463.99
Sum			333,459.66	140,132.03	193,327.63

**Table 2:** Small high temperature anomalies statistics.

Number	Coordinate		Area (m^2^)	Property	Surface temperature range(°C)
	Longitude(E)	Latitude(N)			
1	110° 10′ 37.52″	39° 16′ 2.51″	1,366.90	1^–2^ coal pile, open fire	103.0–225.0
2	110° 10′ 44.66″	39° 15′ 59.46″	331.65	1^–2^ coal pile, open fire	50.2–101.6
3	110° 11′ 1.62″	39° 15′ 45.76″	4,968.13	Gangue	36.6–89.6
4	110° 11′ 0.88″	39° 15′ 36.76″	3,092.70	Gangue	46.0–102.5
5	110° 10′ 28.67″	39° 15′ 24.91″	1,346.92	2^−2^ coal seam, open fire	30.2–253.6
6	110°10′17.08″	39° 15′ 17.99″	10,480.00	Gangue	36.4–103.0
7	110° 10′ 10.65″	39° 15′ 16.26″	3,401.00	Gangue	45.7–153.8

Coal fire zone I is a long northwest–southeast strip along the overhanging wall of the pit, ranging from 45 to 225 m wide and approximately 816 m long. There are four roadway openings, six open fires and four collapses with many fissures and cracks, which emit smoke and gas. The area of the fire zone is 33,521.64 m^2^, the area of the fire transition zone is approximately 42,113.32 m^2^, and the total area of this coal fire is approximately 75,634.96 m^2^.

Fire zone II corresponds to fire zone I and is is also a long northwest-southeast strip along the overhanging wall of the pit, 30–253 m in width, 1,225 m in length; there are eight open flames and four subsidence events with smoke emissions. The area of the fire zone is 49,760.82 m^2^, the fire transition zone is 94,087.27 m^2^, and the total area of the coal fire is 143,848.09 m^2^.

Fire zone III is composed of two bay-shaped areas III-E and III-W, separated by 80 m sidewalks piled with gangue. The roadways at the bottom of the two areas are connected, and the roof of the coal seam is burnt rock. The fire zone width is 45–135 m, and its length is 260–340 m, with two laneway entrances; there are ten open flames, and three collapses with smoke emerging from the cracks around the areas of subsidence. The area of the fire zone is 45,276.08 m^2^, the fire transition zone is approximately 22,663.05 m^2^, and the total area of this coal fire is 67,939.13 m^2^.

Fire zone IV is a long northwest-southeast strip along the overhanging wall of the pit, ranging from 40–155 m in width and 545 m in length, with three laneway entrances, one collapsed spot and six open flames. Many fissures and cracks emit smoke and gas. The area of the fire zone is 11,573.49 m^2^, the fire transition zone is approximately 34,463.99 m^2^, and the total area of the coal fire is 46,037.48 m^2^.

In pace with eliminating the underlying coal, the overburden subsides and the air conducts into the burning area and hot gas escape from there though the tension cracks, which promotes combustion. As time going, coal burns deeper into the mountain slope, leading to the overlying rocks to gradually subside into the burned-out void^10^. Therefore, the direction of fire advance is from the fire zone to the fire transition zone.

TIR can detect the location of coal fire based on surface signatures^46–48^ but cannot be seen into the subsurface, so the true range of the subsurface burning region cannot be delineated merely from this technique. It is successful to identify and delineated the surface fires with depths less than 10 m, but hard to identify fires deeper than 30 m, because it is need a long time (approximately a decade) to conduct the heat to the surface^35^. Therefore, remote sensing is predominant in revealing near-surface fires but has difficulties identifying fires at greater depths^26,49^.

**Figure 6:**
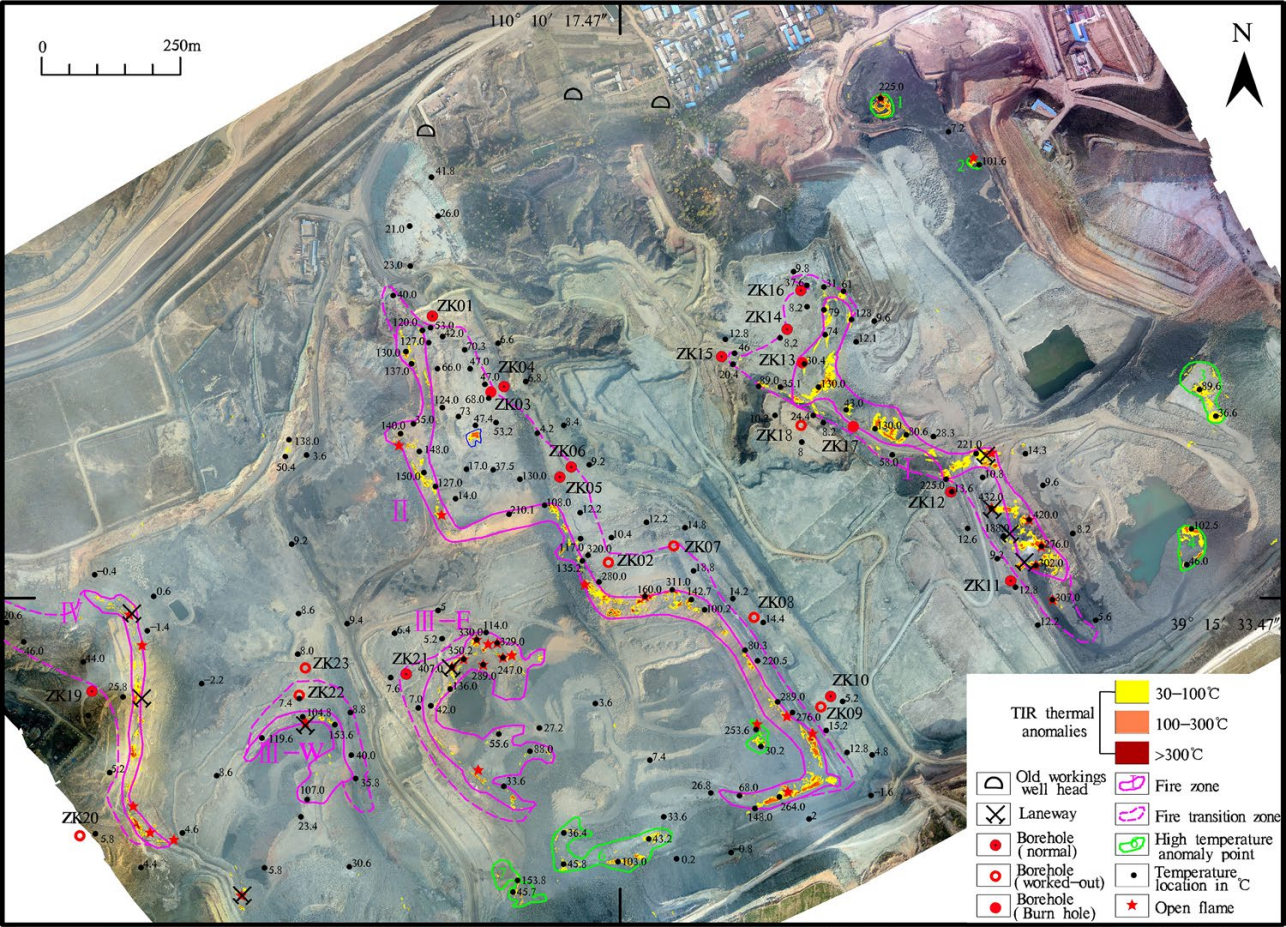
Temperature measurement and drilling location (Black dots represent temperature measurement location with °C; Red solid circles represent holes drilled into burnt rock, red open circles represent the holes drilled into worked-out areas or fissures, red circles with black dots represent the holes drilled into non-fire areas).

Delineation of the subsurface fire is essential to extinguish fire project, including surface subsidence and temperatures, cracks and fissures. Investigation of these variables can approximately identify the areal extent of the fire^25^. Field geological work includes the investigation of fire areas, crack, smoke, laneways and old kiln wellheads, and the measurement of surface temperatures. Ground real information about coal fires in the HCM has been acquired from using portable thermometers. A field survey was conducted during the month of November 2019 to obtain results for validation. Temperature were measured at different heights in the opencast mine to comprehend the connection between the thermal anomalies due to subsurface coal fires and background temperatures (Fig. 6). The high-temperature points and cracks are basically located in the coal fire zone and effectively determine the coal fire range.

### Drilling

**Figure 7:**
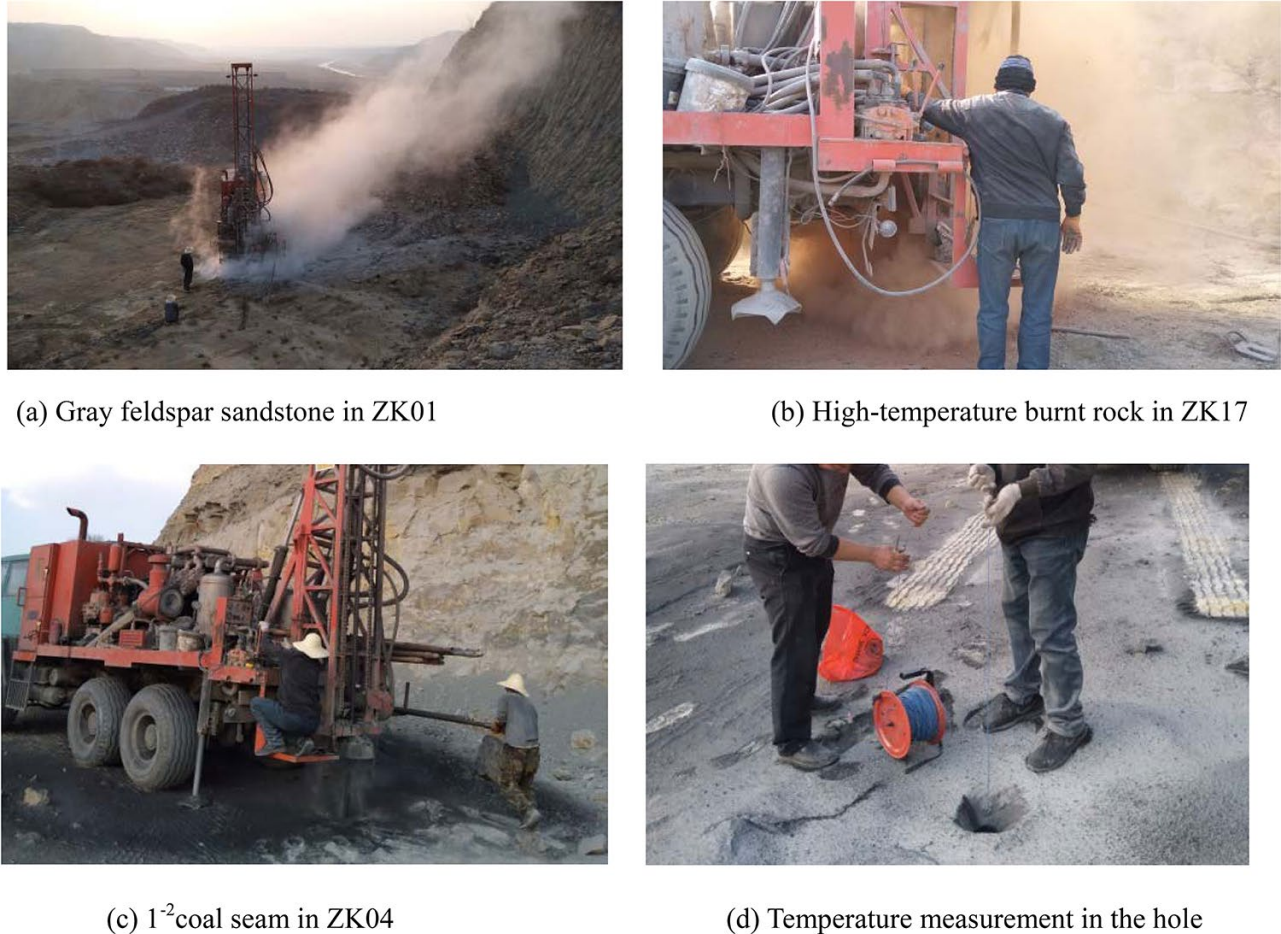
Drilling and temperature measurement in the hole.

Based on the preliminary delineation of the coal fire zone, the centre of the fire zone is determined by deploying drill holes. A corresponding bore is placed on each side of the fire transition zone and the non-fire zone. One borehole is drilled inside first; if it is a high-temperature hole, drilling continues outside; otherwise, drilling is stopped. Using a drill, the lithologies are determined by the characteristics of fragments carried by the wind pressure, such as sandstone, burnt rocks and coal (Fig. 7a, b, c). Temperature is measured from top to bottom every 5 m through the borehole (Fig. 7d), which can approximately verify and modify the boundaries of coal fires. Figure 6 shows the drilling locations, and Table 3 shows the drilling characteristics. Red solid circles represent holes drilled into burnt rock with high temperature, red open circles represent holes drilled into worked-out areas or fissures and red circles with black dots represent the holes drilled into non-fire areas with low temperature.

**Table 3:** Drilling characteristics.

Bore no	Latitude	Longitude	Altitude (m)	Depth (m)	Temperature (°C) Top–bottom (5 m interval)	Feature
ZK01	39° 15′ 49.47″	110° 10′ 3.69″	1,108.00	37.90	13.8–14.2–14.1–14.6–14.8–15.2–16.5–17.7–18 (37.50 m)	28.60–37.90 m is 1^–2^ coal seams, and the opening is 20 m from the surface fissure of the fire zone, which release smoke and hot gases(53.0 °C)
ZK03	39° 15′ 45.05″	110° 10′ 8.12″	1,109.00	32.50	42.0–63.0–84.0–123.0–175.0–196.0–232.0–240.0 (32.50 m)	29.00 m is 1^–2^ coal roofs and reach worked out area; the hole is 6 m from the surface fissure of the fire zone, which release hot gases(68.0 °C)
ZK04	39° 15′ 45.36″	110° 10′ 9.09″	1,111.00	37.20	20.0–19.8–17.2–18.3–19.2–18.6–18.5–17.5–17.6 (37.2 m)	30.70 m is 1^–2^ coal roofs, ZK03 and ZK04 are 25.65 m apart
ZK05	39° 15′ 40.08″	110° 10′ 13.36″	1,122.00	51.60	35.1–39.3–42.1–46.3–51.8–58.2–71.8–82.6–79.4–55.1–37.6–39.1 (51.60 m)	38.50–49.10 m is 1^–2^ coal, the middle (35.00–40.00 m) of the temperature is high
ZK06	39° 15′ 40.67″	110° 10′ 14.19″	1,125.00	46.90	18.1–18.1–18.1–18.2–18.1–18.1–18.3–17.5–16.6–18.2–88.5 (46.90 m)	43.20 m is 1^–2^ coal roofs, and 45.90 m is worked out area; There are gases at the orifice
ZK07	39° 15′ 36.09″	110° 10′ 21.92″	1,111.00	32.80	16.6–16.8–16.7–16.8–16.9–17.4–40.4–53.8 (32.80 m)	27.40 m is 1^–2^ coal roofs, and 30.80 m is worked out area
ZK08	39° 15′ 31.97″	110° 10′ 28.02″	1,129.00	51.20	29.8–30.0–30.7–31.3–31.4–31.7–32.3–32.2–32–31.8–31.3–29.7 (51.20 m)	45.20 m is 1^–2^ coal roofs, and 49.00 m is worked out area
ZK09	39° 15′ 26.78″	110° 10′ 33.07″	1,147.00	47.00	17.5–16.2–15.7–16.6–19.8–20.5–19.8–20.6–21.3–20.6–25.8 (47.0 m)	42.30 m is fissure (or worked out area)
ZK10	39° 15′ 27.39″	110° 10′ 33.80″	1,147.50	79.90	26.0–26.0–26.0–25.7–26.0–25.9–25.7–25–24.7–24.8–24.3–25.4–26.3–25.8–25.9–22.9–17.4 (79.90 m)	67.12–78.5 m is 1^–2^ coal seams
ZK02	39° 15′ 35.10″	110° 10′ 17.04″	1,104.00	32.90	28.8–32.9–35.3–38.9–40.7–44.5–46.5–50.2 (32.90 m)	24.50 m is 1^–2^ coal roofs, 29.30 m is worked out area
ZK11	39° 15′ 34.21″	110° 10′ 47.22″	1,128.00	51.70	12.7–12.5–12.6–12.2–12.2–12.4–12.7–12.9–14.3–16.6–17.8–17.8 (51.70 m)	38.90–49.40 m is 1^–2^ coal seams
ZK12	39° 15′ 39.38″	110° 10′ 42.67″	1,126.00	51.70	13.1–12.9–12.7–12.6–13.5–13.6–14.2–15.3–18.5–20.1–22.2–22.8 (51.70 m)	40.80–50.80 m is 1^–2^ coal seams
ZK13	39° 15′ 46.87″	110° 10′ 31.44″	1,135.00	18.80	192.0–210.0–212.0–214.0–213.8 (18.80 m)	Borehole collapse and Burnt Rock
ZK14	39° 15′ 48.81″	110° 10′ 30.29″	1,134.50	23.50	44.0–49.0–58.0–64.2–68.8–73.2 (23.50 m)	Backfill area with a large amount of loose crushed stones and gangue, and the color of the stone changes a lot
ZK15	39° 15′ 47.18″	110° 10′ 25.40″	1,143.00	75.20	18.4–18.5–18.5–18.6–18.9–18.1–18–17.7–17.6–17.5–17.4–17.1–16.7–16.4–15.9–20(75.00 m)	64.90–75.20 m is 1^–2^ coal seams, rich in water
ZK16	39° 15′ 51.09″	110° 10′ 31.29″	1,134.66	18.80	34.5–37.2–41.2–40.8–41.0 (18.80 m)	Loose dump, steam at the orifice, cracks below 16.80 m
ZK17	39° 15′ 43.15″	110° 10′ 35.32″	1,123.00	9.40	78.0–160.0–210.0 (9.40 m)	6.20 m see red and hard burnt rocks with rock fragment temperature 135.0 °C
ZK18	39° 15′ 43.21″	110° 10′ 31.40″	1,154.00	79.90	22.5–22.6–22.5–22.7–22.6–22.9–23.4–23.5–23.7–24.5–24.7–25.6–26.6–27.9–28.4–32.5–34.0 (79.90 m)	73.40 m is 1^–2^ coal roofs, 75.20 m is worked out area
ZK19	39° 15′ 27.37″	110° 9′ 38.41″	1,151.00	79.90	14.3–14.6–15.1–15.2–15.9–16.9–18.2–19.7–20.1–21.6–23.3–25.6–28.6–32.5–40.6–48.6–50.5 (79.90 m)	74.40–77.20 m is 1^–2^ coal seams
ZK20	39° 15′ 18.95″	110° 9′ 37.62″	1,154.00	75.20	12.5–13.2–13.1–13.3–13.5–13.4–13.7–14.9–13.4–13.7–14.9–14.3–14.7–15–15.6–15.7(75.00 m)	72.60 m is fissure (or worked out area), 74.20 m is 1^–2^ coal roofs
ZK21	39° 15′ 28.54″	110° 10′ 1.98″	1,069.60	37.60	35.2–39.4–42.2–49.7–62.5–82.9–81.9–48.8–36.6 (37.60 m)	28.20–32.90 m is 2^–2^ coal seam, 4.70 m in thickness
ZK22	39° 15′ 27.24″	110° 9′ 53.96″	1,069.00	28.20	47.4–67.5–70–75.4–125.3–149.6–155.7 (28.20 m)	26.80 m is worked out area
ZK23	39° 15′ 28.83″	110° 9′ 54.42″	1,071.00	32.90	23.5–26.3–31.6–41.6–52.1–52.9–55.6–57.1 (32.90 m)	28.20–30.20 m is 2^–2^ coal seam, 30.20 m is worked out area with water

#### Coal fire zone I

The main coal seam of spontaneous combustion is the 1^–2^ coal. The field investigation finds that the four laneways in the fire zone I have high temperatures of 168.0–432.0 °C and a distance of approximately 50.00 m. Borehole ZK11 is approximately 30.00 m west of fire zone I and has not been drilled into the roadway, although it is above the laneway. It is a low-temperature hole (17.8 °C) and covers approximately 39.00 m of rocks in the 1^–2^ coal roof. ZK12 is approximately 20.00 m from the edge of the high-temperature (225.0 °C) of fire zone I, and it is a low-temperature hole (22.8 °C); the cap rock of the 1^–2^ coal is approximately 40.80 m. With thick overburdens, although the ZK11 and ZK12 holes are relatively close to the fire zone, due to the integrity of the strata and insufficient oxygen supply, the fire does not spread far and may only burn near the laneways. The roadways function as chimneys and only emit a certain amount of smoke. ZK13 is approximately 5 m from the smoke point at the boundary of the fire zone; it is located in the back-filled area and drilled to the red burnt rock and is a high-temperature hole (213.0 °C). Then, proceeding 65.00 m outward, and ZK14 is implemented in the middle of the two bifurcated fire zones; the temperature at the bottom of the hole is 73.2 °C. ZK15 with low temperature controls the boundary of the western bifurcation fire zone, which is approximately 25 m from the surface smoke point (46.0 °C). ZK16 is located in the back-fill area, approximately 10 m from the smoke point (37.0 °C); the maximum temperature in the hole is 41.2 °C, and the eastern boundary of the fire zone can be determined. According to the above information, the TIR anomaly bifurcates on the surface to the east and west and may indicate a single fire under-ground. The ZK17 hole close to the fire zone is only 9.40 m deep and difficult to drill deeper due to the high temperature; at 6.20 m, high-temperature hard burnt rocks appear (210.0 °C). ZK18 is approximately 13 m from a crack without smoke emission and approximately 28.00 m from the crack with smoke (24.4 °C), and the bottom of the hole is 34.0 °C.

#### Coal fire zone II

The main coal seam with spontaneous combustion in the fire zone is the 1^–2^ coal. ZK01 is approximately 14 m from the a surface crack with smoke (53.0 °C), and approximately 31 m from a surface high-temperature point (120.0 °C); the bottom temperature is 18.0 °C, and the borehole is considered a non-fire zone; thus, the range between ZK01 and near-surface cracks can roughly delineate the fire zone boundary, which is consistent with the initial fire zone boundary, so no further holes are implemented. Similarly, ZK03 is approximately 6 m from the surface crack with smoke (68.0 °C) and is a high-temperature hole (240 °C), which is determined to be a fire zone. The corresponding hole ZK04 approximately 25 m from ZK03 is a low-temperature hole (17.6 °C), so ZK03 and ZK04 are judged to mark the borders of the fire zone. ZK05 is approximately 22 m from the overhanging fire zone and approximately 24 m from the surface crack with smoke (79.0 °C), and the temperatures in the hole from bottom to top at 5 m intervals are 39.1, 37.6, 55.1, 79.4, 82.6, 71.8, 58.2, 51.8, 46.3, 42.1, 39.3 and 35.1 (°C); these numbers indicate that the temperatures at the bottom and top of the hole are low and those in the middle are high. The top of the 1^–2^ coal is 1,083.5 m in elevation, and the abnormal temperature (79.4 °C) starts at 1,080.4 m, which is 3 m below the coal roof. The temperature anomaly zone is consistent with the burning depth of the 1^–2^ coal and is related to the baking in the overhanging fire zone. ZK06 with vapour and toxic gas at the orifice is approximately 27 m northeast of ZK05, and the temperature at the bottom of the hole is 88.5 °C. Therefore, ZK05 and ZK06 determine the transition zone, and the true boundary of the coal fire area can be extrapolated a few metres north of ZK06. Due to the topography, ZK07 fails to reach the platform in the fire zone and is drilled into the worked-out area, and the temperature at the bottom of the hole is 53.8 °C, which marks the transition fire zone. ZK02 is approximately 32 m east of the crack at the edge of the cantilevered fire zone. It is drilled to the goaf, and the bottom temperature of the hole is 50.2 °C, which indicates the transition fire zone. ZK08 is approximately 50 m from the fire zone below the overhanging wall and is drilled into the worked-out area. Referring to the adjacent ZK10, the goaf with a temperature of 29.7 °C should be at the top of the 1^–2^ coal. Due to the cap with a thickness of approximately 45 m, the oxygen supply is insufficient, and no combustion has occurred at the bottom. ZK09 is approximately 30 m from the active fire, and owing to the existence of cracks, the target layer 1^–2^ coal is not drilled, and the temperature at the bottom of the hole is 25.8 °C. Therefore, ZK10 is drilled 25 m towards the back side, and the thickness of the 1^–2^ coal is approximately 11.38 m; this is a normal low-temperature hole.

In summary, the fire zone of the coal seam slowly burns into the mountains along the steep walls, and the coal seam that is completely covered does not burn underground due to insufficient oxygen supply. The burning rate of coal seams is mainly related to the thickness of the overlying strata and the development of fractures. The fire is extinguished naturally where the overburden layer is so enough intact and thick that fractures fail to reach the surface to supply more air^10^.

#### Coal fire zone III

The main coal seam with spontaneous combustion in the fire zone is the 2^–2^ coal, which is located in the lowest part of the mining area where the wall overhangs pit. In the eastern fire zone III-E, the overhanging wall shows the 1^–2^ coal with a thickness 1–2 m and ongoing combustion, and the top covers burnt rocks.

ZK21 is approximately 50 m from a crack with hot gas emission, the highest temperature in the hole is 82.9 °C, and the temperature is abnormally high at 1,042–1,052 m on the roof of the coal seam. According to the field survey, the 2^–2^ coal roof is exposed at the bottom of the pit with a few open fires, and it can be inferred that the abnormally high temperature in ZK21 is caused by baking, which results in smouldering. ZK22 is approximately 35 m from the crack with hot gas and smoke in the III-W fire zone and is drilled into worked-out area. Because ZK22 is a high-temperature hole (155.7 °C), ZK23 is implemented at a greater interval. The hole is approximately 50 m from ZK22 and 84 m from a crack at the edge of the fire zone, and it is still drilled to a worked-out area with a temperature of 57.1 °C. ZK22 and ZK33 may be connected via roadways with a cap thickness of approximately 28 m. It can be inferred from the exposed laneway with open fire to ZK23 that the fire extends approximately 100 m along the roadway and that the temperature decreases from 407 °C to approximately 60 °C.

#### Coal fire zone IV

The main seam with spontaneous combustion in the fire zone is the 1^–2^ coal. ZK19 is approximately 35 m from a crack, and the temperature at the bottom of the hole is 50.5 °C. Because the terrain is steep and difficult to reach, ZK20 is approximately 90 m away from the cracks, and the temperature at the bottom of the hole is 15.7 °C, which is a non-fire zone. From the comparison of ZK19 and ZK20, it can be seen that the cantilevered fire zone has a baking heating effect on the coal seams smouldering at a short distance, and the temperatures of the strata far from the overhanging walls tend to be normal.

The above drilling data show that the preliminarily delineated fire area is basically accurate, and only some parts need to be modified.

## Discussion and conclusion

The largest coal consumer, China experiences the most coal fires in the world. Therefore, it is important for China to monitor and execute coal fire evaluation, and suitable suppression work^17^. The government of China has got a clear understanding of this hazard and its impacts on the economy and health, with initiatives for fighting coal fires since 1988. Supposing know the depth of the coal fires, coal fire-fighting teams could fight the fires more successfully and efficiently^50^. To this end, systematic quantification and investigation of actual scenarios of coal seams are always critical issues for the coal fire research community^45^.

Many surface and underground coal fires in northern Shaanxi, such as those in the Longyan, Tanyaoqu, and Huojitu coal mines, are generally less than 10 km^2^, most of them are 2–3 km^2^, and the coal fire distribution is even smaller. In these coal mines, satellite imagery (> 0.5 m) often provides inadequate detail about fissures and coal fire information, and imaging carried out by traditional airborne platforms (< 0.1 m) can provide high temporal and spatial resolutions but with high costs^28,30^. Satellite and conventional platforms are limited in weather, the availability of aircraft, and satellite orbits^32^. Magnetic surveys offer a method for the detection, delineation and monitoring of coal fires^3^, but in the HCM, ground disturbances and destruction block access to coal fires, so the magnetic method cannot effectively delineate the coal fires. Contrast to traditional airborne remote sensing, UAV remote sensing provides fine spatial and higher temporal resolution and low-price to satisfy the critical requirements of spectral, spatial, and temporal resolutions^32,39^. This technique commits to offer the swift and safe survey of thermal areas, often current in dangerous and inaccessible terrain^31^.

As Greene (1969) described previously, it is easy to detect fires less than 10 m in depth on TIR; fires between 10 and 30 m are detected only when the heat is transported to the surface by cracks or is conducted to the surface for several years or more; greater than 30 m in depth, detecting fires at the surface require a decade, or more^35^. In our research, TIR remote sensing technology is very effective in monitoring the high-temperature and thermally anomalous regions formed by surface and near-surface(< 10 m) coal fires, especially in open flame areas. However, it is very troublesome to identify coal fires more than 10 m deep with intact cover; for example, at the positions of ZK5, ZK6, ZK22, and ZK23, with no thermal abnormalities on the TIR image, the borehole temperatures are abnormally high. Therefore, remote sensing interpretation of RGB orthophoto images, ground investigations and drilling are needed to compensate for the shortcomings of TIR images. Surface subsidence, cracks, fissures, hot gas and smoke are all manifestations of the development of coal fires. They are thermally anomalous areas that expand outward from the open flame area and may mark the locations of the next open flame areas.

Our study demonstrates a low cost and effective technique to detect the main coal fires in northern Shaanxi based on UAV remote sensing and provides an accurate basis for fire suppression projects.

## Data Availability

The data and analysis generated during the current study are available from the corresponding author on reasonable request.
